# Cuidados paliativos, cuidados de fin de vida y COVID-19: revisión de alcance

**DOI:** 10.15649/cuidarte.2601

**Published:** 2023-03-31

**Authors:** Oscar Yesid Franco-Rocha, Gloria Mabel Carrillo González, Nathaly Rivera-Romero

**Affiliations:** 1 . University of Texas at Austin, Austin, TX, USA. Email: oyfrancor@unal.edu.co University of Texas University of Texas at Austin USA oyfrancor@unal.edu.co; 2 . Enfermera, Magister en Enfermería, Doctora en Enfermería. Profesora Asociada, Universidad Nacional de Colombia. Bogotá, Colombia. Email: gmcarrillog@unal.edu.co Universidad Nacional de Colombia Universidad Nacional de Colombia Colombia gmcarrillog@unal.edu.co; 3 . Enfermera, Magister en Psicología y Salud Mental. Profesora Asistente, Universidad Nacional de Colombia. Bogotá, Colombia. Email: nriverar@unal.edu.co Universidad Nacional de Colombia Universidad Nacional de Colombia Colombia nriverar@unal.edu.co

**Keywords:** Cuidados Paliativos, Cuidados Paliativos al Final de la Vida, Infecciones por Coronavirus, Pandemias., Palliative Care, Hospice Care, Coronavirus Infection, Pandemics., Cuidados Paliativos, Cuidados Paliativos na Terminalidade da Vida, Infecgóes por Coronavirus, Pandemias.

## Abstract

**Introducción:**

La COVID-19 exacerbó el déficit en la prestación de cuidados paliativos y de fin de vida y aumentó la sobrecarga de los servicios de salud, pero se desconoce la extensión de la literatura sobre dicho tema.

**Objetivo:**

Describir la evidencia sobre la prestación de cuidados paliativos y de fin de vida en adultos durante la pandemia de COVID-19.

**Materiales y método:**

Revisión de alcance según el marco metodológico de Arksey y O'Malley. La búsqueda se realizó en inglés y español; en PubMed, Scielo, la Biblioteca Virtual en Salud, y la base de datos de investigación en Coronavirus. Las publicaciones se filtraron por título, resumen y lectura completa. Los resultados se sintetizaron de acuerdo con la técnica “charting”.

**Resultados:**

Se incluyeron 51 publicaciones. En total emergieron cinco categorías: 1) caracterización de los cuidados paliativos, 2) planificación avanzada de cuidados, 3) acompañamiento a familiares y seres queridos, 4) telesalud, 5) rol de enfermería en los cuidados paliativos.

**Discusión:**

El coste social de la pandemia se refleja en el aumento en la carga de unidades de cuidados paliativos, mayor porcentaje de mortalidad y la disminución de la edad promedio de fallecimiento. Futuros estudios deben abordar el impacto psicosocial en los seres queridos de los pacientes, así como el impacto a nivel comunitario.

**Conclusiones:**

Los cuidados paliativos y de fin de vida constituyen una herramienta fundamental para la atención de pacientes con COVID-19. La pandemia potenció el desarrollo de las tecnologías de la información y las comunicaciones para la prestación de cuidados paliativos.

## Abstract (en)

**Introduction:**

COVID-19 exacerbated the deficit of palliative and end-of-life care provision and increased healthcare services' burden, but the extent of the literature on that topic is unknown.

**Objective:**

To describe the evidence of palliative and end-of-life care provision in adults during the COVID-19 pandemic.

**Materials and methods:**

The scoping review was performed according to Arksey and O'Malley's methodological framework. The search was conducted in English and Spanish, in PubMed, SciELO, the Virtual Health Library, and the Coronavirus research database. The articles were filtered by title, abstract, and full text. The results were summarized according to the charting technique.

**Results:**

Fifty-one publications were included. A total of five categories emerged: 1) Palliative care characteristics, 2) advanced care planning, 3) support for family members and loved ones, 4) telehealth, and 5) nursing role in palliative care.

**Discussion:**

The social cost of the pandemic is reflected in the increased burden of palliative care units, higher mortality rates, and the decreased average age of death. Future studies should address the psychosocial impact of COVID-19 on patients' loved ones, as well as the impact at the community level.

**Conclusions:**

Palliative and end-of-life care is an essential tool for COVID-19 patients care. The pandemic has enhanced the development of information and communication technologies to deliver palliative care.

## Resumo

**Introdujo:**

A COVID-19 exacerbou o déficit na prestagáo de cuidados paliativos e em fim de vida e aumentou a sobrecarga sobre os servidos de saúde, mas a extensáo da literatura sobre tal tópico é desconhecida.

**Objetivo:**

Descrever as evidencias sobre a prestagáo de cuidados paliativos e de fim de vida em adultos durante a pandemia da COVID-19.

**Materiais e Método:**

Revisáo do escopo de acordo com a estrutura metodológica de Arksey e O'Malley. A pesquisa foi realizada em ingles e espanhol; PubMed, Scielo, a Biblioteca Virtual de Saúde e o banco de dados de pesquisa do Coronavirus. As publicares foram filtradas por título, resumo e texto completo. Os resultados foram sintetizados de acordo com a técnica gráfica.

**Resultados:**

51 publicares foram incluidas. No total, surgiram cinco categorias: 1) caracterizado dos cuidados paliativos, 2) planejamento de cuidados avanzados, 3) acompanhamento de parentes e entes queridos, 4) telesaúde, 5) papel de enfermagem nos cuidados paliativos.

**Discussao:**

O custo social da pandemia se reflete no aumento da carga sobre as unidades de cuidados paliativos, maiores taxas de mortalidade e uma diminuido da idade média de morte. Estudos futuros devem abordar o impacto psicossocial sobre os entes queridos dos pacientes, bem como o impacto a nivel comunitário.

**Conclusóes:**

Os cuidados paliativos e de fim de vida sáo uma ferramenta fundamental para o cuidado de pacientes com COVID-19. A pandemia tem melhorado o desenvolvimento das tecnologias de informado e comunicado para o fornecimento de cuidados paliativos.

## Introducción

Los cuidados paliativos tienen como objetivo mejorar la calidad de vida de las personas diagnosticadas con enfermedades potencialmente mortales^1^. Estos resultados son más favorables si el tratamiento se inicia de manera temprana^2^, pues se optimiza el manejo de síntomas, se mejora la relación con el equipo de salud y se reduce la utilización de los servicios de salud^3^.

Calvache y cols.^4^ reportaron que el número de personas que requieren cuidados paliativos ha aumentado, especialmente en los grupos de personas más jóvenes. La Organización Mundial de la Salud (OMS) ha estimado que anualmente 40 millones de personas requieren de cuidados paliativos. Sin embargo, más del 70% de estas viven en países de bajos y medianos ingresos^1^. Para el caso específico de América Latina, hay un total de 922 servicios en toda la región, lo que significa 1.63 servicios/unidades/equipos de cuidados paliativos por cada millón de habitantes, aunque la mayoría de países ofrecen cuidados paliativos, estos servicios se brindan de manera aislada^5^.

La pandemia de COVID-19 causó alteraciones en el funcionamiento de los sistemas de salud en todo el mundo^6^. No obstante, dado el pronóstico de la enfermedad, los cuidados paliativos son un elemento esencial en el abordaje de personas afectadas por la COVID-19^7^. En Colombia se ha promovido la accesibilidad de estos servicios7, pero desde antes de la pandemia existían deficiencias en la prestación de cuidados paliativos4, las cuales se han exacerbado debido a la crisis sanitaria^8^. Otras revisiones han analizado el sufrimiento, la planificación de cuidados paliativos y cómo integrarlos durante la COVID-19^9^^,^^10^. No obstante, se desconoce la extensión de dicho tema en aspectos como el uso de herramientas digitales y el rol de enfermería. Teniendo en cuenta lo anterior, nuestro objetivo es describir la evidencia disponible sobre la prestación de cuidados paliativos y de fin de vida en adultos durante la pandemia de COVID-19 con el fin de entender la extensión y naturaleza del tema. La pregunta de investigación que orientó la búsqueda fue: ¿cuál es la evidencia disponible sobre la prestación de cuidados paliativos y al final de la vida en adultos durante la pandemia de COVID-19?

## Materiales y Métodos

Se empleó una revisión de alcance, así: 1) identificación de pregunta de investigación, 2) identificación y selección de publicaciones, 3) “charting” los datos, y 4) síntesis y reporte de resultados, consistente con la metodología de Arksey y O'Malley^11^ y PRISMA^12^^,^^13^.

La búsqueda se realizó entre marzo y abril de 2021 en las bases de datos PubMed, Scielo, Biblioteca Virtual en Salud (BVS) y la base de datos de investigación en Coronavirus. Se creó la siguiente ecuación con términos MeSH: “covid-19 and palliative care and end-of-life care and nursing care”. Se incluyeron publicaciones de los años 2020 y 2021, en idiomas inglés y español. Se excluyeron las publicaciones que describían los cuidados paliativos y al final de la vida en población pediátrica y los estudios que no se desarrollaron en el contexto de la pandemia. La presente revisión no cuenta con un protocolo de búsqueda registrado.

Inicialmente, se identificaron publicaciones por título. Posteriormente se filtraron por resumen y lectura completa. La información se extrajo en una base de datos que contenía información sobre los autores, año y tipo de publicación e información sobre la prestación de cuidados paliativos y de fin vida. Los resultados se sintetizaron de acuerdo con la técnica “charting” de Ritchie y Spencer^14^, la información se agrupó y categorizó en temas clave de acuerdo con su similitud. Aunque no se disponía de un formato calibrado para la síntesis de datos, los autores revisaron por separado y en conjunto las categorías.

## Resultados

**Figura 1:**
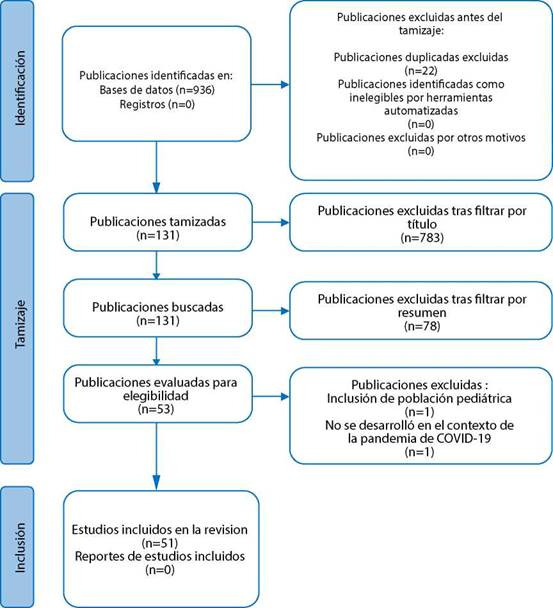
Proceso de búsqueda y selección de publicaciones.

La búsqueda arrojó un total de 936 publicaciones. Tras eliminar obras duplicadas (n= 22), filtrar por título, resumen y texto completo, 51 publicaciones conformaron la muestra final (Figura 1).

*Fuente. Adaptado de Page y cols.*^15^

El 62,75% de las obras provenían de PubMed, el 33,33% de la BVS y el 3,92% de Scielo y la base de datos de investigación en coronavirus (1.96% cada una). El 90,10% de las publicaciones se encontraron en inglés y el 88,67% se publicó durante el 2020. La mayoría fueron artículos descriptivos (35,29%), por cajas de herramientas y programas para la atención (13,72%), recomendaciones de expertos (11,76%), comentarios (9,80%), revisiones (9,80%), cartas al editor (7,84%), editoriales (5,88%), reportes de caso (3,94%) y opiniones (1,97%).

Emergieron cinco categorías: caracterización de los cuidados paliativos, planificación avanzada de cuidados, acompañamiento a familiares y seres queridos, telesalud y rol de enfermería en los cuidados paliativos.

### Caracterización de los cuidados paliativos

Durante los primeros meses de la pandemia se registró un aumento en el número de referencias a cuidados paliativos^16^ y de pacientes hospitalizados en hospicios^17^. El porcentaje de remisiones de pacientes con COVID-19 a unidades de cuidados paliativos varió entre el 30%^18^, 40%^19^ y 60%^20^. Las causas de remisión más frecuentes fueron cuidados de fin de vida^16^^,^^18^^,^^21^. Las personas con COVID-19 tuvieron menos días de cuidado paliativo, pues tendían a fallecer más rápido^16^^,^^17^^,^^19^.

El porcentaje de fallecimiento de los pacientes con COVID-19 remitidos a cuidados paliativos estuvo entre el 74%^21^ y el 80,6%^19^. En general, la edad promedio de fallecimiento fue más baja en pacientes con COVID-1 9^16^^,^^17^^,^^20^^,^^22^, la mortalidad fue más alta en hombres^18^^-^^20^^,^^22^^-^^24^ y en promedio estos pacientes reportaron más de 3 comorbilidades^18^^,^^19^^,^^21^, siendo las enfermedades cardiovasculares las más _frecuentes_^16^^,^^19^^-^^21^^,^^25^_._

La disnea fue el síntoma más común^18^^,^^19^^,^^21^^,^^26^^,^^27^, seguido por la agitación/ansiedad^18^^,^^19^^,^^21^^,^^26^, las secreciones de las vías respiratorias altas^26^^,^^27^ y la somnolencia^16^^,^^21^. No obstante, pueden presentarse síntomas vagos, difusos y atípicos^27^. Eriksen y cols.^27^ recopilaron síntomas adicionales que se pueden presentar en los pacientes con COVID-19 en cuidados paliativos, como delirio, debilidad, somnolencia, entre otros.

La mayoría de los pacientes contaba con infusiones por vía subcutánea para el manejo de síntomas^16^^,^^18^^,^^19^^,^^21^^,^^27^. Los medicamentos administrados fueron principalmente opioides y sedantes para el manejo de la disnea y la agitación, respectivamente^16^^,^^19^^,^^21^^,^^26^^,^^27^. Cuando el paciente presentaba delirio se le administraban antipsicóticos^18^.

### Planificación avanzada de cuidados

La planificación avanzada de cuidados constituye una herramienta que le permite a los pacientes y a sus familiares plantear objetivos de cuidado^28^^,^^29^ que orienten a los equipos de salud en la atención que el paciente desea recibir^23^. También evita las hospitalizaciones, tratamiento en unidades de cuidados intensivos^30^^-^^33^ y de sostenimiento de vida^27^^,^^28^^,^^32^^-^^34^ no deseados. Por otro lado, aunque el paciente y su familiar tengan la intención de recibir tratamientos para el sostenimiento de la vida, la pandemia ha generado situaciones éticas complejas con respecto a la distribución de recursos^29^^,^^32^^,^^35^^,^^36^ en especial en momentos donde los servicios de salud se encuentran saturados, lo que podrá requerir de estrategias como el triage para orientar la distribución y aliviar la carga de los servicios de salud^30^. Brindar esta información al paciente y sus familiares, y analizar todos los posibles resultados, les permite establecer objetivos de cuidado de manera objetiva, autónoma y aterrizada^30^^,^^37^^-^^39^.

Dado que la enfermad de COVID-19 se ha descrito como impredecible y de rápido deterioro^31^^,^^32^^,^^40^, la planeación de cuidados debe hacerse de manera temprana^22^^,^^32^^,^^37^^,^^41^. En personas consideradas de alto riesgo es ideal planificar los cuidados antes de que sea necesario hospitalizarlos^25^^,^^42^ o tan pronto reciben el diagnóstico de COVID-19^22^^,^^33^^,^^38^^,^^41^. La planeación de cuidados debe hacerse de manera rutinaria, pues las preferencias pueden cambiar^27^^,^^28^.

Si bien abordar estos temas es complicado, la COVID-19 tiene el potencial de fomentar estas discusiones^25^^,^^32^^,^^37^^,^^43^. En casos donde el paciente ha sido diagnosticado con disfunción cognitiva se debe hacer la planificación de cuidados junto con el pariente más cercano^27^. Se deben describir las preferencias del paciente de manera explícita en la historia clínica y compartir esa información con el equipo tratante^30^^,^^33^^,^^44^. Cuando sea posible, el paciente debe indicar quien será su representante legal^45^.

Los cuidados de fin de vida deben mantener la dignidad del paciente y proveer confort. Consecuentemente, algunos autores recomendaron suspender las intervenciones que causen dolor^27^. También propiciar un ambiente tranquilo (por ejemplo, apagar alarmas e instaurar medidas de monitoreo menos estresantes^46)^.

### Acompañamiento a familiares y seres queridos

Las restricciones de acompañamiento y la inmovilidad incrementan la sensación de aislamiento de los pacientes y el distrés en las familias^24^^,^^35^^,^^47^, por lo que debe fomentarse la comunicación y, de ser posible, el acompañamiento presencial, en especial cuando se acerca el final de la vida^27^^,^^28^'^31^'^33^'^38^^,^^46^'^48^^-^^50^.

La muerte tiene diferentes significados para cada individuo^51^. Debido a las medidas de aislamiento para la prevención y control de la pandemia ritos de duelo se suspendieron, lo que puede generar estrés, duelo anticipado y duelo complicado^22^^,^^24^^,^^25^^,^^31^^,^^50^^,^^51^. El duelo complicado puede generar pensamientos intrusivos, preocupación, alienación de relaciones sociales, y percepción de pérdida de la vida. Teniendo en cuenta lo anterior, a los familiares y seres queridos de los pacientes que fallecen en el contexto de la pandemia, sobre todo a quienes fallecen de COVID-19, debe brindárseles apoyo durante el duelo^28^.

### Telesalud

Las restricciones instauradas para el control de la pandemia promovieron un modelo de atención basado en la telesalud^40^^-^^42^^,^^48^^,^^51^^-^^56^. Las herramientas tecnológicas fomentaron la comunicación con pacientes, familiares y entre profesionales de la salud, mientras se minimizaba el riesgo de exposición al virus^39^^,^^41^^,^^47^^,^^48^^,^^57^^,^^58^. También facilitó el monitoreo y tratamiento de síntomas^25^^,^^52^^,^^55^, la prestación de servicios de manera más consistente y directa^52^^,^^53^, el establecimiento de objetivos de cuidado^46^^,^^52^^,^^55^, la toma de decisiones^48^^,^^55^ y el apoyo espiritual^41^.

Algunas de las barreras reportadas fueron los vacíos legales y financieros para la prestación de servicios de manera digital^53^, el difícil establecimiento y mantenimiento de rapport^42^^,^^55^ y confianza^42^, la imposibilidad de analizar el lenguaje no verbal^53^^,^^55^ la sobrecarga en los trabajadores de la salud^47^ y la comunicación con personas que no son hábiles o no tiene acceso a herramientas digitales^42^^,^^53^. Finalmente, aunque algunos familiares y seres queridos estaban dispuestos a usar la videoconferencia para participar en la planeación de cuidados^55^^,^^59^^),^ también indicaron que preferían estar junto a sus seres queridos, por lo que se estas estrategias deberían ser empleadas con cautela^59^.

### Rol de enfermería en los cuidados paliativos.

Las enfermeras paliativistas alrededor del mundo están brindando soporte a los equipos de emergencia, unidades de cuidado intensivo y servicios de larga estancia^36^^,^^41^^,^^44^^,^^60^^,^^61^. No obstante, hay vacíos en la evidencia frente al rol de estos especialistas en escenarios comunitarios^60^. Adicionalmente, el personal de estas áreas generalmente ha recibido poco o nulo entrenamiento en la prestación de cuidados paliativos^56^^,^^61^. Por otro lado, se refirió que algunos especialistas en paliación tienen dificultad para confiar en personal temporal o nuevo frente a la prestación de cuidados paliativos y de fin de vida^43^.

Es necesario educar y entrenar a enfermeras y otros profesionales de la salud en la prestación de cuidados paliativos para que estos puedan proveer atención a aquellos que están cerca del final de la vida^22^^,^^28^^,^^43^^,^^46^^,^^61^^-^^63^. Algunos programas digitales han demostrado efectividad y factibilidad en dicho aspecto^64^. De otra parte, para facilitar la atención paliativa los especialistas pueden crear guías parael manejo de síntomas^29^^,^^39^^,^^61^^,^^62^ y apoyo psicosocial^29^^,^^39^. Sin embargo, es importante que los equipos de cuidados paliativos estén disponibles para asesorar de manera remota en el manejo de casos serios y refractarios^34^^,^^39^^,^^61^.

Los enfermeros especialistas en cuidado paliativo lideran la coordinación de cuidado psicosocial, cultural y espiritual de los pacientes y su familia60. Además, están entrenados en la comunicación para el establecimiento de objetivos de cuidado, consideraciones clínicas éticas, manejo de síntomas y cuidado en el final de la vida^41^. No obstante, este tipo de especialistas es escaso^34^^,^^39^^,^^46^ y puede experimentar diversos retos en situaciones de crisis y contingencia, como cambios en la razón enfermera-paciente, escasez de insumos médicos y dificultades de comunicación por el uso de barreras de protección adicionales^18^^,^^34^^,^^35^^,^^41^^,^^57^^,^^65^.

Rogers y cols.^35^ reportaron que más del 80% de los profesionales de la salud identificaron un aumento en las necesidades emocionales de sus pares. Esto puede deberse a la sobrecarga de trabajo, cambios en los patrones de sueño y alimentación^40^^,^^58^, estrés^49^, el duelo de los profesionales^31^ y temor al rechazo por parte de la comunidad^58^.

La evaluación de los pacientes debe hacerse de manera regular, concisa y rápida^65^ para anticipar situaciones de crisis^26^^,^^43^. De otra parte, se deben fortalecer las relaciones con los pacientes para promover su confianza en los profesionales de enfermería^45^^,^^66^. Frente a los cuidados no farmacológicos se destacaron los cambios de posición, el cuidado de la cavidad bucal y el uso de elementos para regular la temperatura^27^. También se reportaron adaptaciones en las instalaciones físicas como inclusión de ventanas en las puertas e instalación de teléfonos inteligentes en las habitaciones para mejorar la valoración del paciente^46^. El tratamiento farmacológico está sujeto a los síntomas que experimente el paciente; así pues, los opioides y sedantes son los de primera elección para el cuidado paliativo.

Finalmente, enfermería debe conocer y entender qué síntomas pueden ser tratados de manera remota y cuáles requieren atención de manera presencial^41^. El Reino Unido propuso el uso de diferentes vías de administración (oral, rectal o sublingual)^56^^,^^65^ y la administración de medicamentos por parte del cuidador familiar en respuesta a la escasez de profesionales de la salud^56^. Para este último caso, los cuidadores familiares deben ser bien entrenados, pues si el paciente fallece después de la administración de un medicamento, el cuidador familiar puede sentirse culpable del desenlace de salud^56^.

## Discusión

En este estudio se presenta la evidencia disponible sobre la prestación de cuidados paliativos y cuidados al final de la vida en adultos durante la pandemia de COVID-19. Tras el análisis de los resultados se identificó un aumento considerable en la carga de las unidades de cuidados paliativos^16^. Por su lado, el aumento en el porcentaje de mortalidad de los pacientes^19^^,^^21^ y la disminución en la edad promedio de fallecimiento^16^^,^^17^^,^^20^^,^^22^ pueden reflejar el coste social de la pandemia. Los síntomas más frecuentes en los pacientes con COVID-19 en cuidados paliativos son la disnea, la agitación y las secreciones respiratorias^18^^,^^19^^,^^21^^,^^26^^,^^27^. Esto podría constituir una diferencia frente al resto de la población en cuidados paliativos, que refieren el dolor como uno de los síntomas más frecuentes^1^. Por su parte, se observa la importancia de la planificación avanzada de cuidados como una estrategia útil para la prestación de servicios^23^ y que propende por el respeto de los deseos de los pacientes y sus familiares^27^^,^^28^^,^^30^^-^^34^, consistente con otras revisiones^9^^,^^10^.

Debe promoverse la comunicación entre los pacientes y sus seres queridos, sobre todo cuando estos se encuentran en la etapa de fallecimiento^27^^,^^28^^,^^31^^,^^33^^,^^38^^,^^46^^,^^48^. Frente a este aspecto, las tecnologías de la información y las comunicaciones promovieron la comunicación entre pacientes y familiares^39^^,^^41^. También fue esencial para la continuidad de cuidados, el monitoreo y tratamiento de síntomas^25^^,^^52^^,^^55^.

A pesar de esto, barreras como la imposibilidad de interpretar la comunicación no verbal^53^^,^^55^, el acceso a internet^42^ y el desconocimiento frente al manejo de herramientas digitales^53^ puede generar disparidades en salud. Estas barreras pueden ser mayores en países en vías de desarrollo. Por ejemplo, en Colombia solo 7.6 millones de personas tienen acceso a internet, siendo los residentes de las zonas rurales aquellos con menor acceso a internet (0.13 personas por cada 100 habitantes^67^.

A pesar de que una de las fortalezas de esta revisión es la inclusión de publicaciones recientes, el estudio de la COVID-19 ha avanzado de manera acelerada, lo que puede generar algunos cambios en el abordaje de los pacientes con esta condición. Por su parte, la inclusión de publicaciones independientemente de su tipología puede constituir una ventaja, pues puede reflejar el conocimiento y la experiencia de los profesionales y pacientes desde diferentes perspectivas. No obstante, también representa una limitante dado el rigor de las publicaciones, aspecto que no fue evaluado en esta revisión. De igual manera, implementar algunas recomendaciones puede ser complicado en países de ingresos bajos y medios, como Colombia. Futuros estudios deberán abordar el impacto psicosocial de los cuidados paliativos y de fin de vida en familiares y seres queridos, así como el impacto a nivel comunitario causado por los cambios en los procesos de duelo teniendo en cuenta el contexto Latinoamericano.

## Conclusión

Los cuidados paliativos y de fin de vida constituyen una herramienta fundamental para la atención de pacientes con COVID-19. Los resultados recopilados en la presente revisión muestran patrones demográficos sobre la recepción de cuidados paliativos durante la pandemia (p.e., sexo y edad). Frente a la planificación de cuidados, las publicaciones incluidas indican que debe hacerse de manera conjunta entre pacientes, seres queridos y equipos de salud considerando los recursos disponibles y la situación de salud pública a nivel local, regional y nacional.

Debe fortalecerse la educación y entrenamiento de los profesionales de enfermería para la prestación de cuidados paliativos, independientemente de su campo de acción. La pandemia evidenció que incluso las enfermeras comunitarias pueden enfrentarse a situaciones donde la prestación de cuidados paliativos a los pacientes y sus seres queridos es fundamental. No obstante, el desconocimiento les genera ansiedad.

Finalmente, la pandemia potenció el desarrollo y avance de las tecnologías de la información y las comunicaciones para la atención en salud. A su vez, permitió la identificación de algunos aspectos a mejorar, como el acceso equitativo y la interpretación de la comunicación no verbal, aspectos que requieren mayor estudio con el fin de garantizar la calidad en la atención.
